# *In Vitro* Model of Tumor Cell Extravasation

**DOI:** 10.1371/journal.pone.0056910

**Published:** 2013-02-20

**Authors:** Jessie S. Jeon, Ioannis K. Zervantonakis, Seok Chung, Roger D. Kamm, Joseph L. Charest

**Affiliations:** 1 Department of Mechanical Engineering, Massachusetts Institute of Technology, Cambridge, Massachusetts, United States of America; 2 Department of Biological Engineering, Massachusetts Institute of Technology, Cambridge, Massachusetts, United States of America; 3 School of Mechanical Engineering, Korea University, Seoul, Korea; 4 Charles Stark Draper Laboratory, Cambridge, Massachusetts, United States of America; Massachusetts Institute of Technology, United States of America

## Abstract

Tumor cells that disseminate from the primary tumor and survive the vascular system can eventually extravasate across the endothelium to metastasize at a secondary site. In this study, we developed a microfluidic system to mimic tumor cell extravasation where cancer cells can transmigrate across an endothelial monolayer into a hydrogel that models the extracellular space. The experimental protocol is optimized to ensure the formation of an intact endothelium prior to the introduction of tumor cells and also to observe tumor cell extravasation by having a suitable tumor seeding density. Extravasation is observed for 38.8% of the tumor cells in contact with the endothelium within 1 day after their introduction. Permeability of the EC monolayer as measured by the diffusion of fluorescently-labeled dextran across the monolayer increased 3.8 fold 24 hours after introducing tumor cells, suggesting that the presence of tumor cells increases endothelial permeability. The percent of tumor cells extravasated remained nearly constant from1 to 3 days after tumor seeding, indicating extravasation in our system generally occurs within the first 24 hours of tumor cell contact with the endothelium.

## Introduction

Tumor metastasis is the hallmark of malignant cancer and the cause of 90% human cancer deaths [1], [2]. Thus the real threat of cancer is that malignant tumor cells are able to escape from the primary site and form metastatic colonies in secondary sites. During metastasis, epithelial cancer cells undergo epithelial-mesenchymal transition (EMT), disperse from the primary tumor, and intravasate into the vascular system. Cancer cells, once in the circulation, are transported to a remote site where they can extravasate from the vascular system into the surrounding tissue to colonize at remote sites, completing the dissemination process [3], [4]. While there exists an enormous literature on oncogenic transformation and emergence of the primary tumor, much less research addresses issues related to metastasis [5]. There is little doubt that a deeper understanding of cancer metastasis could lead to novel therapeutic strategies targeting the invasion pathways and improving cancer survival rates [6].

Extravasation is a vital step in cancer cell dissemination, which enables successful establishment of a secondary metastasis. The process of extravasation consists of: 1) transport via blood circulation, 2) arrest adjacent to a vessel wall, and 3) transmigration across the endothelial monolayer into the secondary site [7]. For tumor cell arrest on vessel wall, two possible modes have been proposed. One, proposed by Paget as the “seed and soil” hypothesis, is that tumors of different organs show unique patterns of metastatic colonization to specific organs through site-selective adhesion [8]. In a second mode, tumor cells become trapped in small vessels due to size restriction as tumor cells tend be larger than other circulating cells and can also aggregate with platelets [9], [10], [11]. While both modes have been observed during extravasation [3], [12], [13], [14], it is still not clear which is dominant or whether different tumor types preferentially exhibit a particular type of arrest prior to transmigration. Furthermore, invasive behavior of tumor cells depends on cross-talk between tumor and host cells in a complex three dimensional (3D) microenvironment [15]. Direct observation of tumor cell arrest on an endothelium with controlled microenvironmental conditions would provide useful insight into this crucial step of extravasation. Also the establishment of secondary metastases at a distant organ after transmigration requires tumor cell interaction with a diverse array of extracellular matrix (ECM) components, such as collagen, laminin and fibronectin [16]. However, the roles of microenvironmental cues and cytokine gradients within the tissue during the process of extravasation are not well understood.

Conventional studies of extravasation rely primarily on tail-vein injection of tumor cells with subsequent imaging and analysis *in vivo* [17], [18]. Although these *in vivo* experiments provide the most physiologically representative conditions for extravasation, they have limitations in studying tumor and vessel interactions as videomicroscopy provides only limited visualization of the event, and tightly-regulated parametric studies are not possible. *In vitro* models offer solutions to these problems, which led to widespread use of the Boyden chamber for simulating the invasion or migration of cancer cells [19], [20]. The relative simplicity of operation is an advantage of this system, but there are limitations in using it for studying complex interactions between cancer cells and the endothelium. The Boyden chamber has limited control over the local microenvironment and less than optimal imaging capabilities. In an attempt to address these needs, there has been a growing interest using microfluidic technology since it provides a simple yet effective means to investigate these phenomena under tight control of the biochemical and biophysical environment [21], [22], [23], [24].

We have previously reported an *in vitro* microfluidic platform that offers the capability to more realistically mimic the 3D *in vivo* situation in a controlled environment while simultaneously providing *in situ* imaging capabilities for visualization, thereby enabling quantification of cell-cell and cell-matrix interactions [25], [26], [27], [28]. Moreover, the system enables parametric study of multiple factors in controlled and repeatable conditions as well as study with multiple cell types with an endothelial barrier [26], [29], [30]. While no *in vitro* systems can fully replicate the *in vivo* situation, microfluidics offers the opportunity to create organ-specific microenvironments to explore the different metastatic patterns of different cancer types in a regulated, and easily-visualized model.

Microfluidic platforms of various designs have been previous employed to study cell migration and tumor cell intravasation [24], [31]. In this paper, we used the established microfluidic system to investigate the critical steps of cancer extravasation – tumor cell adhesion to the endothelium, transmigration across the endothelial monolayer, proliferation in remote tissues – and its consequences. Our experimental platform mimics the tumor microenvironment, allows for high resolution imaging of tumor cell extravasation and early steps of colonization, thus enabling better quantification of the critical metrics of cancer cell invasiveness.

## Materials and Methods

### Microfluidic System

**Figure 1:**
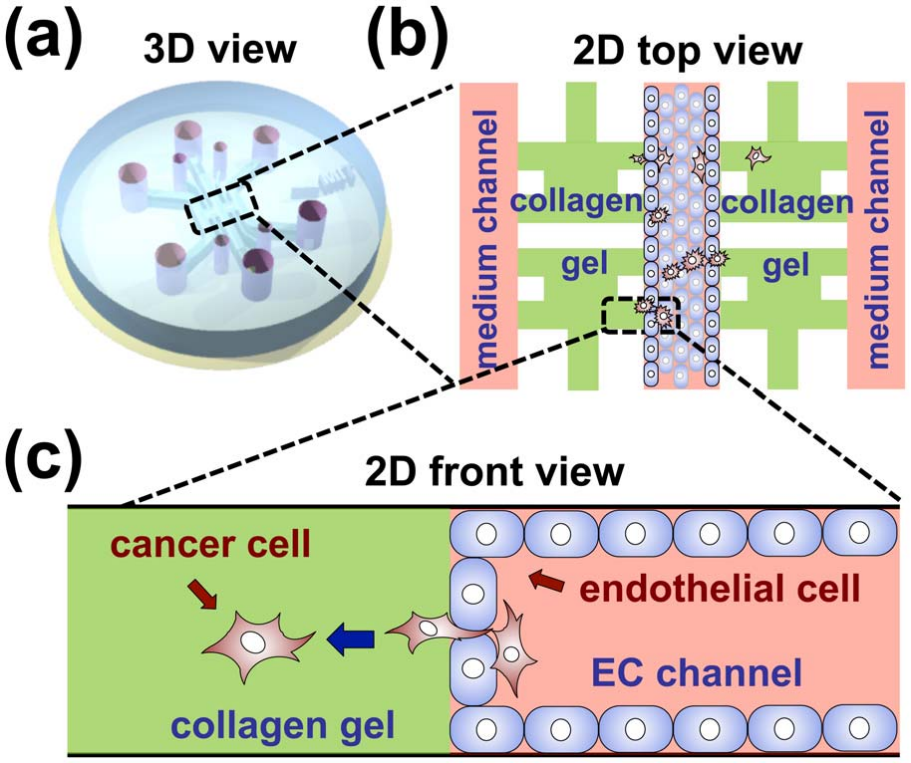
General schematic of the device. Microfluidic system consisting of three independently addressable media channels, separated by chambers into which an ECM-mimicking gel can be injected (a). Figure 1b shows the inside view of the device with endothelial monolayer (blue) covering the center channel. This channel acts as cell channel where both endothelial cells and cancer cells are introduced to form monolayer and transmigrate respectively (b). The green region indicates the 3D space filled with collagen gel and the pink regions indicate the channel filled with medium. Cancer cells which adhere to endothelial monolayer can extravasate into the collagen gel region as shown in (c).

In these studies we used a previously developed microfluidic system consisting of three independently addressable media channels, separated by chambers into which an ECM-mimicking gel can be injected (Fig. 1a). Details of the design and the steps required for fabrication of the systems in PDMS have been described previously [25], [28], [30]. In brief, the microfluidics system consists of molded PDMS (poly-dimethyl siloxane; Silgard 184; Dow Chemical, MI) through which access ports are bored and bonded to a cover glass to form a microfluidic channels. Channel cross-sectional dimensions are 1 mm (width) by 120 µm (height). The PDMS layer is formed from a patterned SU8 photoresist on a silicon wafer using soft-lithography. To enhance matrix adhesion, the PDMS channels are coated with a PDL (poly-D-lysine hydrobromide; 1 mg/ml; Sigma-Aldrich, St. Louis, MO) solution. Next, collagen type I (BD Biosciences, San Jose, CA, USA) solution (2.0 mg/ml) with phosphate-buffered saline (PBS; Gibco) and NaOH is injected into the gel regions of the device via 4 separate filling ports using a 10 µl pipette and incubated for 30 min to form a hydrogel, chosen to represent ECM in 3D space. When the gel is polymerized, endothelial cell medium is immediately pipetted into the channels to prevent dehydration of the gel. Upon aspirating the medium, diluted Matrigel™ (BD science) solution (3.0 mg/ml) is introduced into the cell channel and the excess Matrigel™ solution is washed away 1 minute later using cold medium. 2D top and face views of the device are shown in Fig. 1b–c to show how this microfluidic system is used to model extravasation. Endothelial cells are first introduced to cover the entire middle channel and later cancer cells are introduced so they adhere to and transmigrate across the already formed endothelium into the gel region. The middle channel acts as a cell channel where both endothelial cells and cancer cells are introduced to form a monolayer and transmigrate, respectively.

### Cell Culture and Cell Selection

Human microvascular endothelial cells (hMVECs) were commercially obtained (Lonza) and cultured in endothelial growth medium (EGM-2MV, Lonza). Cells were cultured in standard culture flasks and the medium was changed every two days until seeding. During the seeding process, 40 µl of hMVEC suspension at 2×10^6^ cells/ml was introduced into the prepared microfluidic device. The cells were kept in a 37°C incubator for 1 hour to allow the adhesion of cells to the collagen scaffold wall. All experiments were conducted using hMVECs of passage 8 or lower. Human mammary adenocarcinoma cells (MDA-MB-231) were chosen due to their invasiveness and their ability to metastasize *in vivo* [32], [33]. A GFP-expressing version of the MDA-MB-231 cell line (provided by F. Gertler, MIT) enabled live-cell imaging via fluorescent microscopy. Cancer cell lines were cultured in standard DMEM media (Sigma) with 10% fetal bovine serum (Invitrogen) and antibiotics. The human mammary epithelial cell line MCF-10A (provided by Brugge Lab, Harvard Medical School) was cultured as described previously [34]. Two days after endothelial cell seeding, tumor cells were introduced into the same channel where endothelial cells had formed a complete monolayer. Culture flasks containing the tumor cells were first washed with PBS and the cells were later trypsinized for 5 min to make the cell suspension in cancer cell medium. For seeding, 40 µl of 50000 cells/ml tumor cell suspension medium was placed in one side of the channel reservoir and left to equilibrate. The tumor cell suspension medium in the channel was removed 1 hour later and all channels in the device were filled with endothelial cell culture medium. Control experiment with MCF-10A was done following the exact tumor cell seeding protocol. All cultures were kept in a humidified incubator, which was maintained at 37°C and 5% CO_2_.

### Permeability of Endothelial Monolayer

Upon formation of a complete endothelial monolayer by day 2, the diffusive permeability was measured with fluorescently-labeled dextrans in culture medium as shown in Fig. S1 (10 kDa cascade blue and 70 kDa MW Texas red, Invitrogen). The endothelial monolayers grown in our microfluidic system exhibited lower diffusive permeability values for the smaller molecular weight dextran confirm the presence of a size-selective endothelial barrier. To characterize changes in permeability upon extravasation, we used the 70 kDa dextran. Before introducing dextran into the device, the endothelium was first examined using a phase contrast microscope (Nikon, Tokyo, Japan) to confirm monolayer formation on both the top and the bottom of the channel by focusing at different heights. All medium in the device reservoirs was aspirated first and later re-filled with control medium in the side channels whereas the cell-seeded middle channel was filled with fluorescent dextran solution (10 µg/ml) in medium in the cell-seeded middle channel. Precisely 110 µl was promptly added to each channel so as to maintain equal pressures and thereby avoid convective flow across the hydrogel. Devices were then placed in the incubator for 3 hours to reach steady state, fluorescent images of dextran distributions were taken using an epi-fluorescent microscope (Nikon TE300, Hamamatsu ORCA-ER camera) and processed using OPENLAB 4.0.4 software. Images were later analyzed using MATLAB to calculate fluorescence intensity across the monolayer. To determine the diffusional permeability, we calculated the distribution of fluorescence intensity change as a function of distance perpendicular to the plane of the endothelial layer. A detailed procedure for measuring permeability has been described previously [24], [35], [36], [37]. Briefly, we used the equation P = D [dC/dx]/ΔC_ec_ where P is the diffusive permeability (cm/s), dC/dx is the gradient of the dextran concentration, ΔC_ec_ is the concentration difference across the monolayer, and D is diffusion coefficient of dextran.

### Immunofluorescent Staining and Image Acquisition

All cells in the device were washed with Phosphate Buffered Saline (PBS) and later fixed with 4% paraformaldehyde for 15 min. After washing twice with PBS, cells were permeabilized with 0.1% Triton-X 100 solution for 5 min and blocked with 5% BSA solution for 5 h. VE-cadherin was labeled with rabbit polyclonal antibody (polyclonal; Alexis Biochemical) at 1∶100 dilution and subsequently applied fluorescently-labeled secondary antibody. Cell nuclei were stained with DAPI (Invitrogen) at 1∶1000 dilution. All images were obtained using a confocal microscope (Leica) and processed with IMARIS software.

### Metrics for Extravasation

Quantitative cell counting was performed after immunofluorescent staining. Confocal data were analyzed using IMARIS and its tracking algorithms for selecting and counting for nuclei in the specific region of interest (ROI). The ROI was the 3D gel region between a PDMS post and the wall as seen in boxed area of Fig. 1b that was selected during confocal imaging and contained both the endothelial lining channel region as well as the collagen gel. ROIs were selected such that edge effects associated with PDMS walls and posts were avoided. The dimensions of the ROI were 250 µm×250 µm×120 µm (height) and each microfluidic device contained total eight ROIs. While each ROIs were analyzed individually, the extravasation percentage was measured per device. As the tumor cells express GFP, cells with both green and blue signal were counted to track the number of tumor cells.

### Statistics

All values reported are averages of measurements from a minimum of 4 devices, each with a minimum of 2 and maximum of 8 ROIs with standard errors. The comparisons between unpaired groups were assessed using unpaired Student’s t-test and the nonparametric Mann-Whitney U statistic whereas paired permeability measurements were assessed using a paired t-test. Tumor seeding density statistics were obtained using one-way ANOVA. Statistical significance was assumed for p<0.05. All tests were performed with SigmaPlot v.12.

## Results and Discussion

### Modeling the Extravasation Process

Although there remains considerable uncertainty regarding the critical, rate-limiting step in the formation of metastatic tumors, the ability of circulating tumor cells (CTCs) to adhere to and transmigrate across the endothelium at a remote site is certainly essential. Numerous studies have addressed this issue, but the challenges of constructing a meaningful *in vitro* testing platform has been a strong impediment to improved understanding, and as importantly, has posed a barrier to the identification of drugs that could inhibit extravasation. Recent studies have begun to address this need using advanced microfluidics [21], [22], [23], but each is has its limitations. In the current model, we demonstrate the capability of monitoring the entire process of extravasation. Our previous studies in a similar system have demonstrated changes in endothelial permeability are closely associated with intravasation [24], so we sought to study similar changes that might occur during extravasation. In addition, by tracking the cells over time, we were able to explore the time-dependent behavior, an important factor that impacts both the survival of the CTCs prior to extravasation as well as their ability to reconfigure the immediate microenvironment prior to transmigration.

### Confirmation of Endothelial Layer Integrity

**Figure 2:**
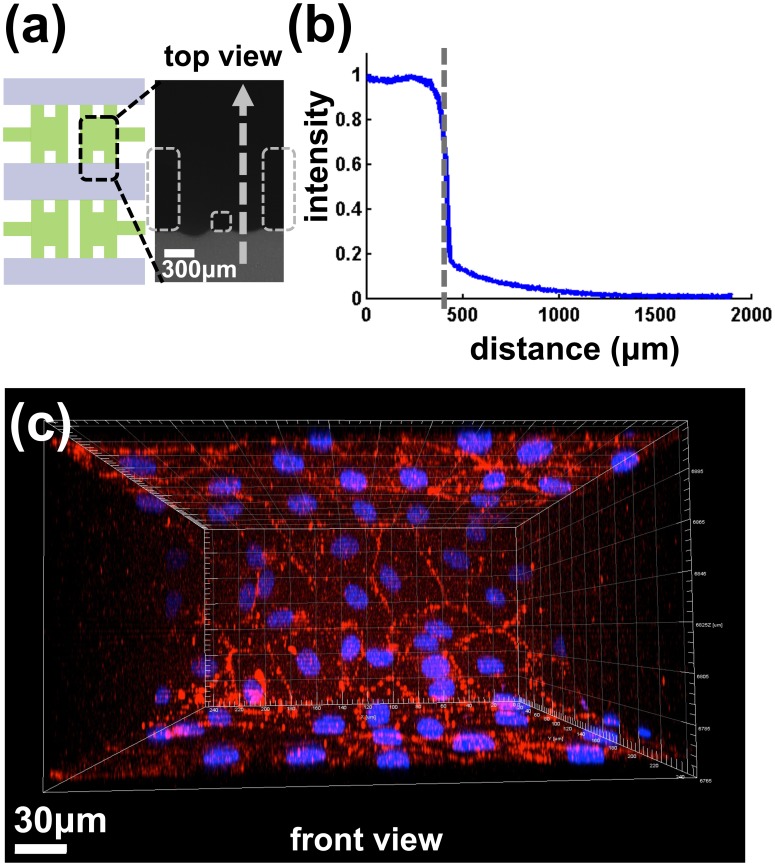
Confirmation of endothelial monolayer integrity. The integrity of the endothelial monolayer was confirmed by both fluorescence imaging of the dextran distribution and confocal microscopy of fixed and labeled cells. An intact endothelial monolayer gives rise to an abrupt intensity drop between the channel and the gel region once the fluorescently-labeled dextran is introduced. Three hours after dextran injection, a sharp drop in fluorescence intensity is seen across the endothelial layer demonstrating its function as a barrier to macromolecules (a). Fluorescence intensity is quantified using Matlab (b). The dashed arrow in (a) the location and direction for the quantification.The intensity value drops to 15% of is peak value due to the barrier effect. The endothelial monolayer is located near the 400 µm point on the plot (shown with dashed line). Samples fixed on the third day after cell seeding and stained for VE-cadherin and nuclei (DAPI-blue) exhibit well-defined junctions with no apparent gaps in the confluent monolayer (c). The confocal image shows the front view of the microfluidic device.

The microfluidic system was designed to model tumor cell extravasation where the tumor cells are introduced into a channel lined with a confluent endothelial monolayer. Using phase contrast microscopy, hMVECs were observed forming a confluent monolayer on the microchannel surfaces and ECM-endothelial channel interface two days after endothelial cell seeding. The integrity of the endothelial monolayer was confirmed by both fluorescence imaging of the dextran distribution and confocal microscopy of fixed and labeled cells. An intact endothelial monolayer gives rise to an abrupt intensity drop between the channel and the gel region once the fluorescently-labeled dextran is introduced, and persists over time as dextran slowly diffuses across the monolayer into the gel (Fig. 2a–b). Samples fixed on the third day after cell seeding and stained for VE-cadherin and nuclei (DAPI-blue) exhibit well-defined junctions with no apparent gaps in the confluent monolayer (Fig. 2c). Quantification and analysis of fluorescence intensity yields values for the endothelial permeability to a 70 kDa dextran (3.70±0.59)⋅10^−6^ cm/s, or roughly one order of magnitude higher than published *in vivo* values but consistent with previously reported values in *in vitro* systems [35], [38]. The higher values of permeability may be due to a variety of factors present *in vivo* but missing from the *in vitro* model. For example, it is well known that the presence of pericytes helps to establish the low permeability of vessels *in vivo* [38]. In view of our previous work demonstrating that increased permeability correlates with increased rates of intravasation [24], to the extent that cells use similar mechanisms for extravasation as intravasation, the present extravasation rates may be viewed as being biased toward higher values than physiologic.

### Optimization of Tumor Cell Seeding

**Figure 3:**
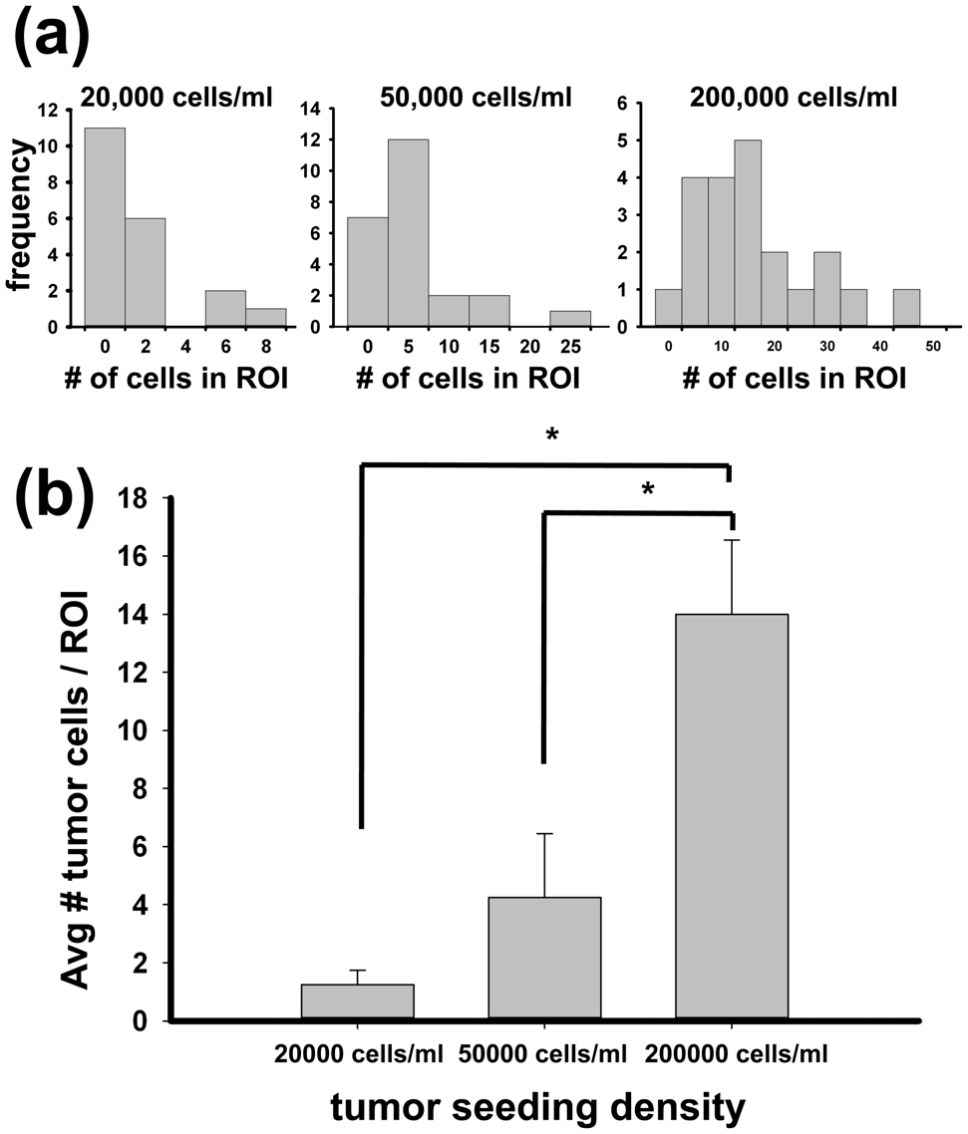
Optimization of tumor cell seeding density. The tumor cell seeding density was optimized to have only a limited number of tumor cells in ROI while maintaining as many experimental ROIs as possible that contain at least one tumor cell so tumor cell events can be observed. Histograms of number of total tumor cells present in each ROI (250 µm×250 µm×120 µm) show different trends in distribution of tumor cells for three different tumor seeding densities: 20,000 cells/ml, 50,000 cells/ml, and 200,000 cells/ml (a). The average value and the histogram can be used for choosing the optimal tumor seeding condition (b). Seeding density of 50,000 cells/ml was chosen as a compromise between mimicking the low number of tumor cells of the *in vivo* of extravasation condition and increasing the chance to have at least one tumor cell to analyze in any given ROI. The statistical significance was tested with one way ANOVA (p<0.05).

From clinical data, the number of tumor cells that have intravasated and travel in circulation has been measured to be less than ∼100 in 7.5 mL of blood on average [39], [40]. For the purpose of these experiments, we chose to use a seeding density which was neither so low we were unable to view a significant number of extravasation events in a reasonable number of experiments, nor so high the tumor cells were densely packed at the endothelial surface. This latter situation might lead to tumor cell interactions that poorly represent the situation *in vivo*. Hence, in this experiment, the tumor cell seeding density was optimized to have only a limited number of tumor cells in the ROI while maintaining as many experimental ROIs as possible that contain at least one tumor cell to facilitate extravasation event observation. Histograms of number of total tumor cells present in each ROI show different trends in distribution of tumor cells for three different tumor seeding densities: 20,000 cells/ml, 50,000 cells/ml, and 200,000 cells/ml (Fig. 3a). Although the smallest tumor seeding density results in the smallest average number of tumor cells in each ROI as shown in Fig. 3b, this is due to having ROIs without any tumor cells 55% of the time. The average value and the histogram can be used for choosing the optimal tumor seeding condition and a seeding density of 50,000 cells/ml was chosen as a compromise between mimicking the low number of tumor cells of the *in vivo* of extravasation condition and increasing the chance to have at least one tumor cell to analyze in any given ROI.

### Extravasation

**Figure 4:**
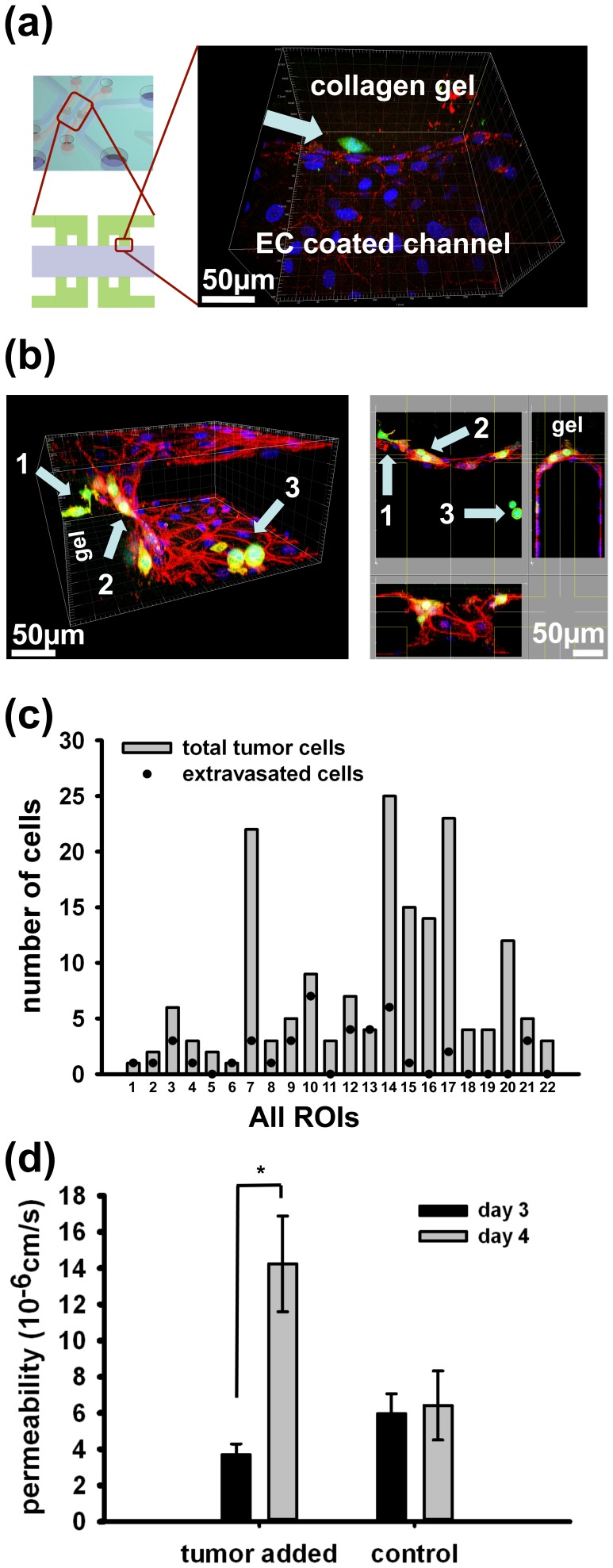
Observation of extravasation and permeability of endothelium. The extravasation event is observed in a sample, which is fixed 1 day after tumor cells are introduced. The region of interest is captured in one confocal image scan and shows one cancer cell (green) that has transmigrated across the endothelium (denoted by VE-cadherin staining in red) and extravasated into the gel region (a). The surface view of the confocal scan shows three different possible locations of tumor cells: 1) extravasated and in gel, 2) adhered and on endothelium adjacent to gel region, and 3) in channel not near the gel. The sectional view of the same confocal scan confirms the different location of the tumor cells (b). The graph shows how many tumor cells have extravasated (dot) among the total tumor cells present (bar) for each region of interest analyzed (c). The tumor cells are categorized as extravasated only when the tumor cells have clearly passed the endothelial monolayer into the gel region. The permeability of endothelial monolayer changes significantly with addition of tumor cells (d). Fluorescently-labeled dextran was introduced on day 3 after endothelial seeding to measure the permeability before tumor and again to same samples on day 4 to see the after tumor seeding effects. The tumor cells are introduced on day 3 after day 3 permeability measurements are taken. The statistical significance was tested with paired t-test (p<0.05).

Tumor cells that disseminate from the primary tumor and survive the vascular system can eventually transmigrate across the endothelium to recolonize at a secondary tumor site. With the microfluidic system developed, we can mimic the extravasation step where tumor cells can transmigrate across an endothelial monolayer into a hydrogel which models the extracellular space of a secondary tumor site. The extravasation event is observed in devices that are fixed 1 day after tumor cells are introduced, and direct quantification of the number of extravasated cells provides a metric of extravasation. The region of interest (ROI) is captured in one confocal image scan and shows one cancer cell, labeled green, that has transmigrated across the endothelium,denoted by VE-cadherin staining in red, and extravasated into the gel region (Fig. 4a). Surface views of the confocal scan from other samples show three different possible locations of tumor cells 1 day after the seeding: 1) extravasated and in gel, 2) adhered and located on endothelium adjacent to gel region, and 3) in the channel not near the gel (Fig. 4b). The surface and sectional views of the device shown in Fig. 4b. All three scenarios of where tumor cells could be are observed here. There are cases where all tumor cells present in the ROI extravasated as well as cases where none of the tumor cells crossed the endothelium. However, it is more common to find regions that contain both extravasated and non-extravasated cells as in Fig. 4b. This is seen quantitatively in Fig. 4c where 51% of ROIs exhibited tumor cells with contrasting fate. The graph shows how many tumor cells have extravasated, as shown by dots, among the total tumor cells present in the each region of interest. The tumor cells are categorized as having extravasated only when they have clearly passed the endothelial monolayer into the gel region.

Measuring permeability of the endothelium is one method for quantifying the quality of endothelial monolayer. In addition, the permeability serves as a metric to quantify the change in endothelium when tumor cells are added to the system and interact via physical attachment to the endothelial surface. Leakiness of the vessel with or without tumor cells provides a possible explanation for events leading to cancer extravasation in that signaling by the tumor CTCs could impair barrier function. Alternatively, the increase in permeability could be a result of tumor cell transmigration. From the present experiments, it is not possible to distinguish between these two scenarios. In this experiment, the permeability changed significantly with addition of tumor cells compared to the permeability change occurring during the same 24 h period when no tumor cells are added (Fig. 4d). Fluorescently-labeled dextran was introduced on day 3 after endothelial cells were seeded to measure the permeability before introducing tumor cells. Images were taken 3 hours after dextran insertion in order to achieve a quasi-steady state. Tumor cells are introduced immediately after the permeability measurements are taken. 24 hours later, fluorescently-labeled dextran was again introduced to measure the permeability after tumor cell – endothelial cell interactions. The initial permeability value was (3.70±0.59)⋅10^−6^ cm/s and the tumor seeding increased endothelial permeability to (14.2±2.6)⋅10^−6^ cm/s (p<0.05) whereas there was no significant change in the control ((6.0±1.1)⋅10^−6^ to (6.4±1.9)⋅10^−6^ cm/s) over the same 24 hour period.

As a control, we measured the change in endothelial permeability and extravasation rates of a normal epithelial cell line, MCF-10A, in our microfluidic system. While extravasation was observed for 38.8±7.9% of the tumor cells in contact with the endothelium 1 day after seeding, the corresponding rate of MCF-10A extravasation across the endothelium was lower, 23.8±4.7%, although not significantly different. Addition of MCF-10A also induced a 2-fold increase in permeability of endothelium from (5.78±0.47)⋅10^−6^ to (11.88±2.15)⋅10^−6^ cm/s (p<0.05) as shown in Fig. S2. This increase is smaller than the permeability increase obtained after the addition of MDA-MB-231 (3.8-fold). Therefore, the MDA-MB-231 cells show an increased tendency to extravasate and compromise endothelial barrier function compared with normal epithelial cells. These results show that our microfluidic system is capable of detecting differences among different epithelial cells lines and our results are consistent with extravasation studies in a transwell assay [41].

The change in permeability of endothelium is regulated by VE-cadherin expression through the Src pathway, and the studies of *in vivo* models have shown that disruption of endothelial barrier function enhanced extravasation efficiency [42]. Several mechanisms exist which could explain changes in permeability due to tumor cell interactions; the permeability increase may also be due to tumor cells locally disrupting endothelial monolayer by contact [43], [44], [45] or through secretion of chemical factors which then compromises the endothelial barrier function [46], but more investigation is needed for clear identification of the cause of the permeability increases.

### Beyond Extravasation

**Figure 5:**
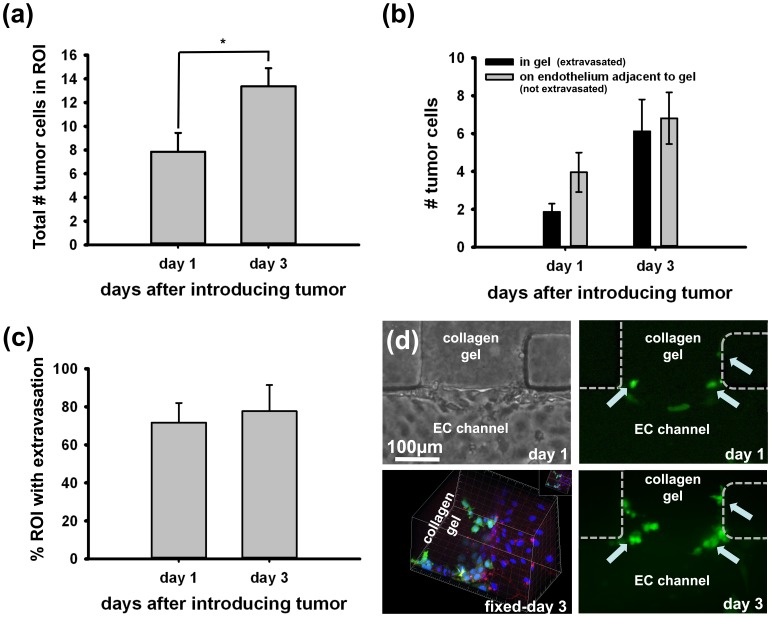
Beyond extravasation. The tumor cell extravasation is observed for up to 3 days after tumor cell seeding and compared to the ones fixed and analyzed on day 1. The total number of tumor cells present in region of interest (ROI) increases significantly from 8 cells on day 1 to 13.5 cells on day 3 while the tumor seeding density as well as other experimental condition remained the same between devices (a). The total number of tumor cells are further subdivided into 2 groups depending on their location, either 1) extravasated and in the gel or 2) adherent to the endothelium adjacent to gel (b). 72% of ROIs exhibited at least 1 extravasated cancer cell (denoted % extravasation occurrence) by day 1 after introducing tumor cells, and 79% of ROIs included extravasation event by day 3, which the difference is not significant (c). The images show number of tumor cell increase (d). The phase contrast image shows the top view of the region of interest on day 1 after tumor seeding. The tumor cells (green) have proliferated from day 1 to day 3 (shown by arrows). The confocal image shows both the tumor cells and endothelium lining. All images are from the same ROI (VE-cadherin: red, nucleus: DAPI-blue, tumor cell: GFP-green).

Tumor cells are observed for up to 3 days after tumor cell seeding and compared to tumor cells on day 1. Average of total number of tumor cells present in ROI increases significantly from 7.9±1.6 cells on day 1 to 13.4±1.5 cells on day 3 while all experimental conditions including the tumor seeding density remained the same (Fig. 5a). This significant increase in number of tumor cells demonstrates proliferation from day 1 to day 3 overall. The total number of tumor cells are further subdivided in Fig. 5b into 2 subgroups depending on their location, either 1) extravasated and in the gel or 2) adherent to the endothelium adjacent to gel. The number of tumor cells per ROI in the gel increased from 1.9±0.4 cells on day 1 to 6.1±1.7 cells on day 3 while the cells on endothelium changed from 4 cells on day 1 to 7 cells on day 3. This increase in tumor cell number from day 1 to day 3 for the extravasated cells could be due to either more cells extravasting over the extra 2 day period, to proliferation, or both. Noting, however, that the percentages of ROIs containing extravasated cells event did not show a significant change for day 1 and day 3 (72% of ROIs exhibited at least 1 extravasated cancer cell by day 1 after introducing tumor cells, and 79% of ROIs included extravasation event by day 3) (Fig. 5c), and assuming the proliferation rates are similar to both populations, this suggests most extravasation events occur within the first day the tumor cells are introduced. This observation is similar in terms of time scale for extravasation seen *in vivo* [17], [47].

### Conclusions

Tumor cells that disseminate from the primary tumor and intravasate into the circulatory system are transported throughout the body. By adhesion to the endothelial wall or plugging in a small capillary, the cells that survive can transmigrate across the endothelial barrier, thus providing a potential nucleating site for metastasis. Our microfluidic platform was applied to study the extravasation of a breast cancer cell line (MDA-MB-231) and their subsequent proliferation in collagen gel, which mimics the 3D nature of the extracellular space. Although microfluidics has limitations in replicating true *in vivo* condition, the system presented here enables a tightly-regulated and well-visualized study of cancer cell extravasation. Using this assay, we have cultured and sustained an endothelial monolayer spanning the entire surface of a microchannel and hydrogel surface, and introduced tumor cells to observe extravasation. We have also quantified the permeability of the endothelial monolayer and showed that endothelial barrier integrity is compromised by the tumor cells. The average number of tumor cells in ROIs increased between day 1 and day 3 after tumor cell seeding while the percentage of ROIs with extravasated cells did not change significantly. These results suggest that extravasation in our system occurs predominantly within the first 24 hours of tumor cell introduction and that proliferation can continue both prior to and after extravasation.

## Supporting Information

Figure S1 **Size selective permeability values of the endothelial monolayer are shown by measurements with10 kDa and 70 kDa fluorescent dextrans.** The smaller sized dextran has a higher permeability value (p<0.05).
(TIF)

Figure S2 **Permeability of the endothelium was measured using fluorescently-labeled dextran to investigate the effect of adding the non-tumorigenic MCF-10A cells (p<0.05).**
(TIF)
