# Supplementary figures and images for: In Vitro Model of Tumor Cell Extravasation
Source: PLoS One. 2013 Feb 20;8(2):e56910. doi: 10.1371/journal.pone.0056910 (PMC3577697; doi:10.1371/journal.pone.0056910)

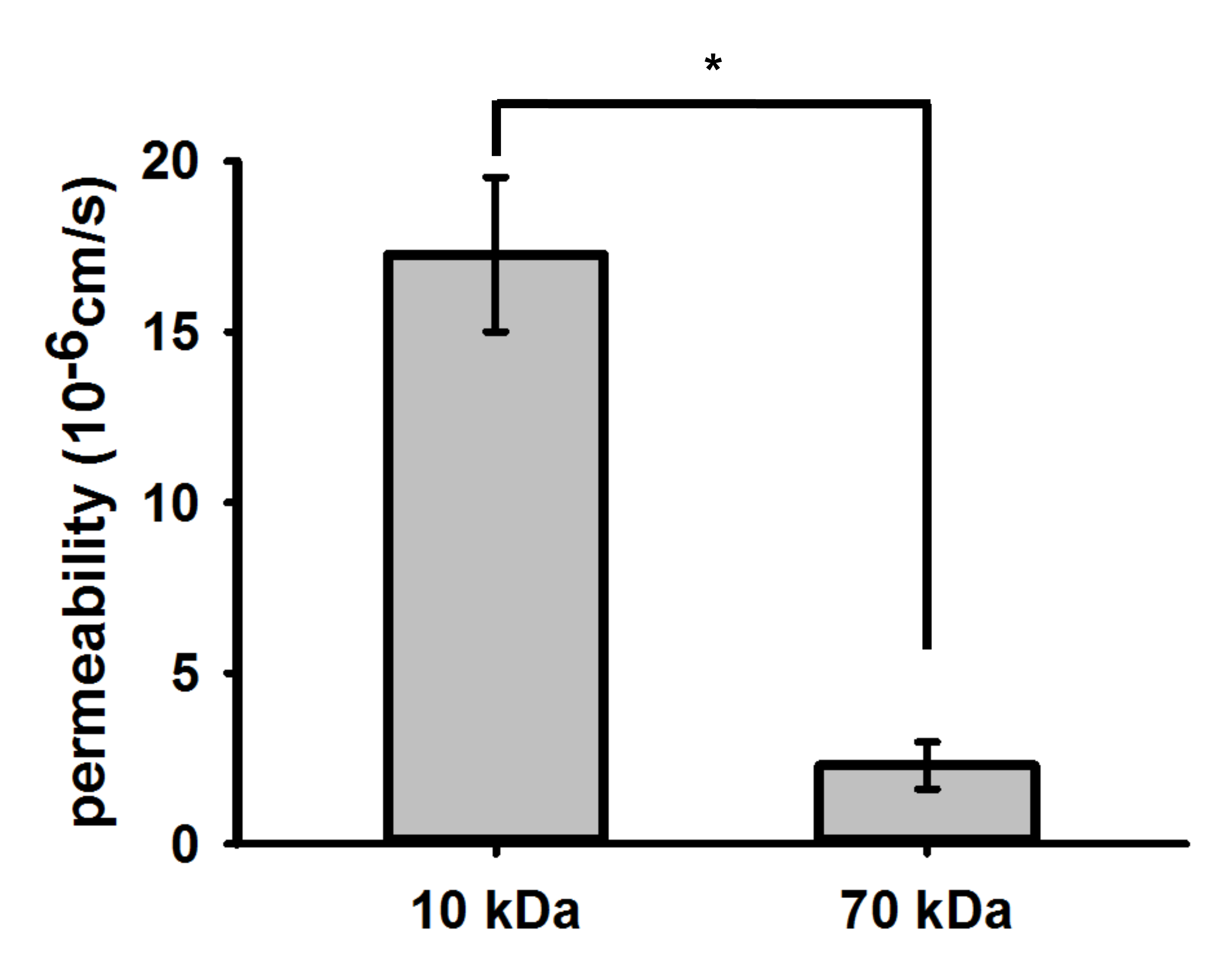

Supplement: Figure S1 — Size selective permeability values of the endothelial monolayer are shown by measurements with10 kDa and 70 kDa fluorescent dextrans. The smaller sized dextran has a higher permeability value (p<0.05). (TIF) [file pone.0056910.s001.tif]

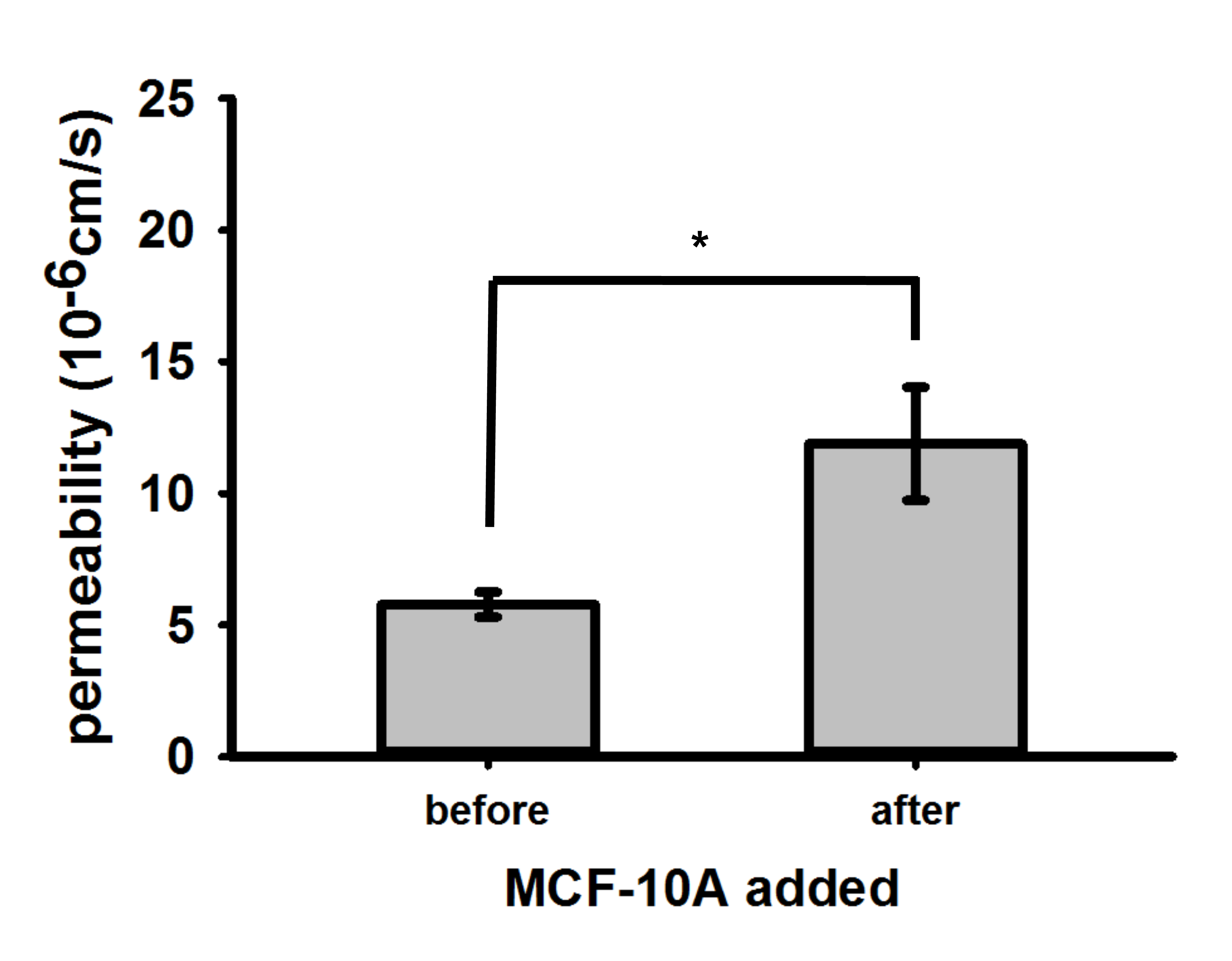

Supplement: Figure S2 — Permeability of the endothelium was measured using fluorescently-labeled dextran to investigate the effect of adding the non-tumorigenic MCF-10A cells (p<0.05). (TIF) [file pone.0056910.s002.tif]
